# Layer‐by‐layer interleukin‐12 nanoparticles drive a safe and effective response in ovarian tumors

**DOI:** 10.1002/btm2.10453

**Published:** 2022-12-01

**Authors:** Antonio E. Barberio, Sean G. Smith, Ivan S. Pires, Sonia Iyer, Ferenc Reinhardt, Mariane B. Melo, Heikyung Suh, Robert A. Weinberg, Darrell J. Irvine, Paula T. Hammond

**Affiliations:** ^1^ Department of Chemical Engineering Massachusetts Institute of Technology Cambridge Massachusetts USA; ^2^ Koch Institute for Integrative Cancer Research Massachusetts Institute of Technology Cambridge Massachusetts USA; ^3^ Whitehead Institute for Biomedical Research Cambridge Massachusetts USA; ^4^ Ragon Institute of Massachusetts General Hospital Massachusetts Institute of Technology Cambridge Massachusetts USA; ^5^ Department of Biology Massachusetts Institute of Technology Cambridge Massachusetts USA; ^6^ Ludwig/MIT Center for Molecular Oncology Massachusetts Institute of Technology Cambridge Massachusetts USA; ^7^ Department of Biological Engineering Massachusetts Institute of Technology Cambridge Massachusetts USA; ^8^ Department of Materials Science and Engineering Massachusetts Institute of Technology Cambridge Massachusetts USA; ^9^ Howard Hughes Medical Institute Chevy Chase Maryland USA; ^10^ Institute for Soldier Nanotechnologies Massachusetts Institute of Technology Cambridge Massachusetts USA

**Keywords:** cancer immunotherapy, cytokine, drug delivery, layer‐by‐layer, nanomedicine, nanoparticle

## Abstract

Ovarian cancer is especially deadly, challenging to treat, and has proven refractory to known immunotherapies. Cytokine therapy is an attractive strategy to drive a proinflammatory immune response in immunologically cold tumors such as many high grade ovarian cancers; however, this strategy has been limited in the past due to severe toxicity. We previously demonstrated the use of a layer‐by‐layer (LbL) nanoparticle (NP) delivery vehicle in subcutaneous flank tumors to reduce the toxicity of interleukin‐12 (IL‐12) therapy upon intratumoral injection. However, ovarian cancer cannot be treated by local injection as it presents as dispersed metastases. Herein, we demonstrate the use of systemically delivered LbL NPs using a cancer cell membrane‐binding outer layer to effectively target and engage the adaptive immune system as a treatment in multiple orthotopic ovarian tumor models, including immunologically cold tumors. IL‐12 therapy from systemically delivered LbL NPs shows reduced severe toxicity and maintained anti‐tumor efficacy compared to carrier‐free IL‐12 or layer‐free liposomal NPs leading to a 30% complete survival rate.

## 1 INTRODUCTION

Immunotherapy has become an increasingly attractive treatment option for cancer therapy since the approval of the checkpoint inhibitor ipilumimab in 2011.^1^ Checkpoint inhibitors have elicited durable complete responses in a broad range of cancers, including some malignancies with previously very poor prognoses.^1^, ^2^ However, checkpoint blockade benefits only a minority of patients in most diseases. It is becoming clear that immunosuppressive or immune excluded “cold” tumor microenvironments (TME) play a key role in nonresponsive tumors.^3^, ^4^ Ovarian cancer is one such malignancy that often presents as a “cold” tumor^5^, ^6^, ^7^ and has been particularly unresponsive to checkpoint inhibition.^8^ One method to bring immunotherapy to such immune excluded environments is to use complementary therapeutics to drive lymphocyte infiltration and activation into tumors while preventing immune system arrest using checkpoint inhibition.^3^

One class of therapeutics with the potential to drive immune infiltration into “cold” tumors are proinflammatory cytokines such as interleukin‐12 (IL‐12), which has shown a potent ability to drive lymphocyte infiltration^9^, ^10^, ^11^ and cure tumors in preclinical models.^12^ However, proinflammatory cytokines tend to be highly toxic when given systemically. Indeed, IL‐12 showed very high, schedule‐dependent toxicity in clinical trials, including two deaths,^13^, ^14^, ^15^ motivating the need for any future IL‐12 therapies to have pronounced spatio‐temporal control over delivery to keep active concentrations in the TME while limiting its systemic exposure. Many newer delivery methods have been attempted to improve IL‐12 therapy, such as gene delivery into the tumor,^16^, ^17^ microparticle delivery^18^, ^19^, ^20^, ^21^ and hydrogel co‐formulations,^22^, ^23^, ^24^ but these approaches are limited by a need for local injection directly into the tumor. This limits the usefulness of such treatments in widely disseminated diseases that do not have easily injectable tumors, such as ovarian cancer which often presents as a disseminated multifocal tumor burden throughout the peritoneal cavity, requiring intravenous or intraperitoneal delivery of therapeutics. Thus, there remains a need for spatio‐temporally controlled, systemically deliverable, nontoxic IL‐12.

One promising route for controlled IL‐12 delivery from a systemically deliverable carrier is the use of an engineered nanoparticle (NP). Systemically administered strategies using simple NP formulations^25^, ^26^ have also been attempted, but have failed to control delivery selectively to the tumor microenvironment or significantly reduce toxicity. However, careful engineering of NP structure and surface chemistry has the potential to eliminate these issues by considering the design criteria for optimal cytokine delivery. For IL‐12, these criteria include (1) high loading and release of active IL‐12, (2) maintenance of NPs on the surface of tumor cells to ensure availability to membrane‐bound IL‐12 receptors on nearby lymphocytes, (3) high association with cancer cells, and (4) decreased systemic exposure and toxicity.

We previously developed a NP delivery vehicle engineered to meet these design criteria using the layer‐by‐layer (LbL) technique to adjust the material properties of the particle.^27^, ^28^, ^29^, ^30^, ^31^, ^32^, ^33^ We showed that a liposomal NP with IL‐12 bound to the liposomal surface and subsequently covered with a bilayer of poly‐l‐arginine (PLR) and poly‐l‐glutamic acid (PLE), termed PLE‐IL‐12‐NP, demonstrated >90% loading efficiency of IL‐12, extended (>24 h) localization on the surface of cancer cells, high selectivity for binding to cancer cells over other cell types, and significant antitumor efficacy when administered intratumorally in multiple subcutaneous tumor models at reduced toxicity compared to carrier‐free IL‐12.^33^ Indeed, we demonstrated that surface binding of the labile cytokine is key for this formulation to avoid the high temperatures, high pressures and sonication required to generate uniform liposomes as well as to generate a high loading efficiency. By using surface linkages on liposomes, we demonstrated loading efficiencies much higher than passive loading techniques in polymer particles and incorporated a chemical linkage that can be further leveraged to control kinetics of release in the future. The PLE coating on these particles is used for active targeting of the particles and payload to the tumor which shows increased activity over other, passively target particles.^28^, ^33^ These particles also critically demonstrate the ability to anchor to the surface of tumor cells due to the PLE surface chemistry^28^, ^33^ which is critical for cytokine activity as compared to more traditional anti‐cancer payloads as cytokines must maintain activity on local immune cells and not cancer cells directly. Moreover, previous work demonstrates the release of active cytokines, a nontrivial finding for labile protein payloads.^33^ These cogent particle designs were demonstrated to be critical for the therapeutic both in previous studies^33^ and in the current work.

**FIGURE 1:**
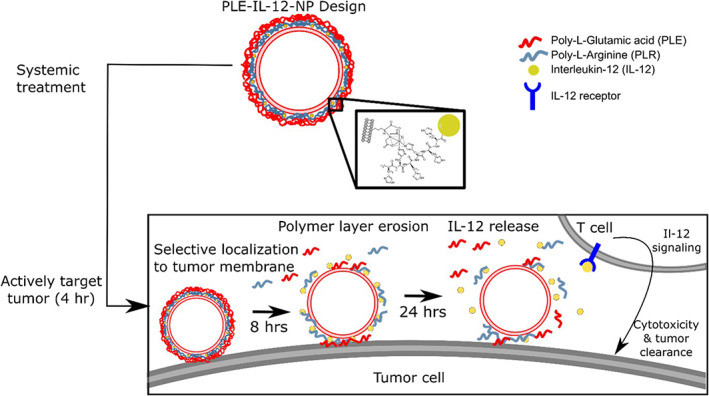
PLE‐IL‐12‐NPs are able to selectively bind to tumor cells and remain localized to cell surfaces, releasing their IL‐12 cargo to activate T cells and other immune cells over a 24‐h period. These characteristics make PLE‐IL‐12‐NPs a strong candidate for safe and efficacious systemic delivery of IL‐12

Achieving systemic delivery of IL‐12, and doing so in a realistic ovarian cancer model, are key achievements necessary to generate a translational therapy. In this work, we hypothesize that PLE‐IL‐12‐NPs can also enable the delivery of IL‐12 to orthotopic ovarian tumors (Figure 1), which requires systemic delivery due to their presentation as widely disseminated metastases throughout the peritoneal cavity. Because our previous work examining nontherapeutic PLE‐layered particles showed association with OVCAR8 ovarian tumors upon systemic administration,^28^ it was hypothesized that PLE‐IL‐12‐NPs will also concentrate IL‐12 in ovarian tumors. The polymer layers act as a hydrated “shield” to minimize off‐target IL‐12 exposure in the blood stream or peritoneal fluid while anchoring the NPs to the surface of cancer cells and releasing active IL‐12 into the tumor microenvironment. In the current study we used the orthotopic HM‐1 and KPCA (an immunologically cold tumor) syngeneic models of ovarian cancer to show that PLE‐IL‐12‐NPs given intraperitoneally or intravenously concentrate IL‐12 within disseminated tumors, increase the therapeutic window of IL‐12, produce long‐term antitumor immune responses, and induce a distinct immunological profile post administration conducive to combination therapy with checkpoint inhibitors. As such we demonstrate the ability to bring the promise of immune treatments to these otherwise refractory tumors by controlling the exposure of toxic, immune infiltrating cytokines within the TME.

## 2 MATERIALS AND METHODS

### 2.1 Particle formulation and characterization

NP formulations were manufactured similar to previous studies.^28^, ^33^, ^34^ Briefly, single chain IL‐12^35^ was produced via vector cloning and expression in Expi293 cells (ThermoFisher Scintific). Liposome cores were made via lipid film drying (rotovap) followed by rehydration and pressure driven extrusion to 50 nm particle size (Avestin Liposofast‐50). Liposomes were comprised of 5% DGS‐NTA (Ni), 65% DSPC, 23.9% cholesterol, and 6.1% POPG by mole for therapeutic NPs. Fluorescent NP were made by lowering DSPC to 60% and adding 5% DOPE for addition of fluorophore. NHS ester fluorophores were added to free amines on DOPE for fluorescently labeled liposomes via overnight reaction at room temperature at pH 8.5 with 5 molar excess dye. Excess dye was removed via tangential flow filtration (TFF). Lipid films were made by drying the indicated lipid mixtures in chloroform by rotovap at 20 mbar for 30 min followed by overnight desiccation under vacuum. 50 nm liposomes were made by first rehydrating films with PBS under sonication at 65°C followed by pressure driven extrusion to desired size (50 nm) at 65°C. IL‐12 was added to extruded particles by overnight incubation under agitation at 4°C. Unreacted IL‐12 was removed and buffer was exchanged to water via tangential flow filtration through a 100 kDa membrane (Repligen). IL‐12 loading was verified by ELISA after digesting particles with 1% triton and 0.1% BSA. Unlayered control particle synthesis ended here. PLE‐IL‐12‐NPs were layered with PLR by mixing with a 0.1 wt eq solution of PLR under sonication, removing unlayered PLR by TFF. PLE was added in at similar manner at 1 wt eq. Polymers were acquired from Alamanda and adsorption conditions were similar to previous report.^33^ Throughout NP manufacture sizes, PDIs and zeta potentials were measured via dynamic light scattering (Malvern ZS90). Nanoparticles were tested for activity in vitro via their ability to stimulate production of IFN‐γ from splenocytes prior to in vivo use. NPs were formulated for systemic injection by mixing 9:1 NP solution:50% Dextrose to make injections isotonic with blood. It is important to note that UL‐NPs and PLE‐IL‐12‐NPs for studies were made from the same batch of UL‐NPs and the layering process results in an approximate increase in diameter of 30 nm and negligible change in final particle charge. Dosing was done on an IL‐12 basis.

### 2.2 Flowcytometry

Antibodies used for immunostaining were against CD69 (biolegend 104545), CD25 (biolegend 102041), NK‐1.1 (biolegend 108753), CD3 (biolegend 100232), CD4 (biolegend 100423), CD8a (BD biosciences 566410), FoxP3 (biolegend 126404), CD45 (biolegend 103112), Ly‐6C (biolegend 128032), Ly‐6G (biolegend 127633), CD274 (biolegend 124331), F4/80 (biolegend 123110), CD11c (BD biosciences 566504), CD11b (biolegend 101217), CD86 (biolegend 105037), and CD103 (biolegend 562722). FoxP3 intracellular staining was carried out using FoxP3 intracellular staining kit (Thermo 00‐5523‐00) following manufacture protocol. Immunostained cells were run on an LSR Fortessa HTS with FACSDIVA software and analyzed using FlowJo V10.5.3.

### 2.3 Cell culture

HM‐1 cells were acquired through Riken BRC. Cells were cultured in α‐MEM, supplemented with 10% FBS and penicillin/streptomycin or as recommended by the supplier. KPCA cell lines^36^ were donated by the Weinberg lab and were cultured in fallopian tube cells media (FT‐media); DME media supplemented with 1% Insulin Transferrin‐Selenium (Thermo Fisher Scientific; ITS‐G, 41400045), 100 μl EGF (10 μg/ml), 4% heat‐inactivated fetal bovine serum (Thermo Fisher Scientific; IFS, F4135) and 1% penicillin and streptomycin. All cells were grown in a 5% CO_2_ humidified atmosphere at 37°C. All cell lines were murine pathogen tested and confirmed mycoplasma negative by Lonza MycoAlert™ Mycoplasma Detection Kit. Lentivirus was used to produce stable production (following 72 h of puromycin selection) of mcherry and luciferase in HM‐1 cells.

### 2.4 Animal studies

All animal experiments were approved by the Massachusetts Institute of Technology Committee on Animal Care (CAC) and were conducted under the oversight of the Division of Comparative Medicine (DCM).

### 2.5 Biodistribution

1E06 HM‐1 mcherry luc2 tumor cells were inoculated in B6C3F1 mice via intraperitoneal injection. Tumors were allowed to establish for 2 weeks. PLE‐IL‐12‐NPs and Unlayered IL‐12 NPs were made following the procedure above with Sulfo‐Cy7 NHS ester dye (Lumiprobe), and confirmed to have equivalent fluorescent properties via plate reader (Tecan). NPs were injected either intravenously via the retro‐orbital route or intraperitoneally at 5 μg doses of IL‐12. Mice were euthanized 4 and 24 h after dosing and liver, kidneys, spleen, and tumors were removed and immediately placed in PBS on ice. Organs were imaged for NP signal (excitation: 745 nm, emission: 800 nm) via an In Vivo Imaging System (IVIS, Perkin Elmer) immediately after harvest. Organs were frozen immediately following imaging and stored at −80°C. Data were analyzed using Living Image software. Background fluorescence measurements were made for each organ based on signal from dextrose only treated mice. Regions of interest (ROIs) were made around treated organs using the contour ROI setting in Living Image. Total radiant efficiencies (TRE) were measured for each treated organ and corrected by the average radiant efficiency from the matching organ in dextrose treated controls. Percent recovered fluorescence for each organ was then calculated as $\frac{\mathrm{TRE}\left(\text{organ}\right)}{\sum_{\text{mouse}}\mathrm{TRE}}$. These % recovered fluorescence values were then normalized by organ weight, similar to previously reported studies.^28^

### 2.6 Cytokine levels in organs

Following biodistribution studies, organs were further processed to extract all protein from individual organs using Miltenyi Biotech gentle MACS Octo Dissociator following recommended protocol for protein extraction. Briefly, organs were placed in M tubes with enough buffer to make a 50 g tissue/ml buffer solution. Buffer used for tissue homogenization was RIPA lysis buffer (Thermofisher #89900) with HALT protease inhibitor cocktail (Thermofisher #78430) and 1% active silicon from Y‐30 emulsion (Sigma) for anti‐foaming purposes. Organs were then homogenized using gentleMACS Octo Dissociator. Samples were spun at 4000 rcf to remove tissue debris and supernatants were analyzed by ELISA for cytokine content.

### 2.7 In vivo toxicity tests

To test toxicity, B6C3F1 mice (Jackson Labs 100010) were injected either intravenously via the retro‐orbital route or intraperitoneally with varying doses as indicated of PLE‐IL‐12‐NPs, dose matched soluble IL‐12, dose matched unlayered NPs or PBS for 5 daily doses and monitored daily for weight change. Serum was collected 3 h after the last dose and assayed for IL‐12 and IFN‐γ levels via ELISA (Peprotech).

### 2.8 In vivo efficacy tests

1E06 HM‐1 mcherry luc2 tumor cells were inoculated in B6C3F1 mice or 1E06 KPCA tumor cells were inoculated in C57BL/6 mice via intraperitoneal injection. Tumors were allowed to establish for 1 week. Subjects were treated with 5 μg intravenously via the retro‐orbital route or 5 μg or 10 μg intraperitoneally of IL‐12 in PLE‐IL‐12‐NPs, Unlayered NPs, or carrier‐free and compared to PBS controls for five daily doses. Mice were weighed daily to track toxicity. Serum was collected after the last dose to test for systemic cytokine levels. Mice were tracked for tumor burden twice weekly via IVIS. Mice were sacrificed based on ascites accumulation and/or overall body condition.

### 2.9 Statistical analysis and data availability

GraphPad PRISM 5 was used to perform statistical analyses. Multiple comparisons were performed using multiple *t* tests, one‐way analysis of variance (ANOVA), or two‐way ANOVA followed by post hoc tests as indicated in figures. The data for this study are available within the article, with additional data available in the Supporting Information.

## 3 RESULTS

### 3.1 PLE‐IL‐12‐NPs are concentrated in tumors upon systemic administration

As a first test of systemic availability and delivery of IL‐12 from PLE‐IL‐12‐NPs, the biodistribution of the NPs was analyzed in HM‐1 tumor bearing mice. For these studies a Sulfo‐Cy7 fluorophore was conjugated to the NP core via an NHS ester linkage to an amine‐carrying lipid head group (1,2‐dioleoyl‐sn‐glycero‐3‐phosphoethanolamine [DOPE]). Animals were treated 14 days after tumor inoculation via intravenous (IV) or intraperitoneal (IP) injection (Figure S1a). Subjects were euthanized 4 h or 24 h after treatment and organs were collected and imaged for NP fluorescence via an *in vivo* imaging system (IVIS) (Figure S1b, S1c, Figure S2). These data demonstrate that the LbL coating plays a critical role in concentrating NPs in the tumors. Both layered and unlayered formulations accumulated in tumors when given IV and IP at both 4 and 24 h time points, however the PLE‐IL‐12 NPs given IP showed tumor accumulation 30% greater than UL‐NPs at 4 h. IV delivery similarly showed greater accumulation with the PLE‐IL‐12‐NPs (a 100% increase over UL‐NPs) although with much less (~10‐fold) accumulation overall compared to IP delivery. Intriguingly, when given IP, the concentration of PLE‐IL‐12 NPs in the tumor even exceeded that found in the liver (Figure S1b, S1c), kidney and spleen (Figure S3) by 7‐fold, 17‐fold, and 4‐fold respectively. Indeed, PLE‐IL‐12‐NPs showed less accumulation in the liver (Figure S1c ) in both delivery routes in comparison to UL‐NPs. While tumor accumulation is important, accumulation alone is not enough to generate a therapeutic response. We previously demonstrated^33^ that PLE coated particles showed higher cell association with tumor cells compared to their unlayered counterparts. This increased association coupled with increased localization shows potential for improved therapy.

**FIGURE 2:**
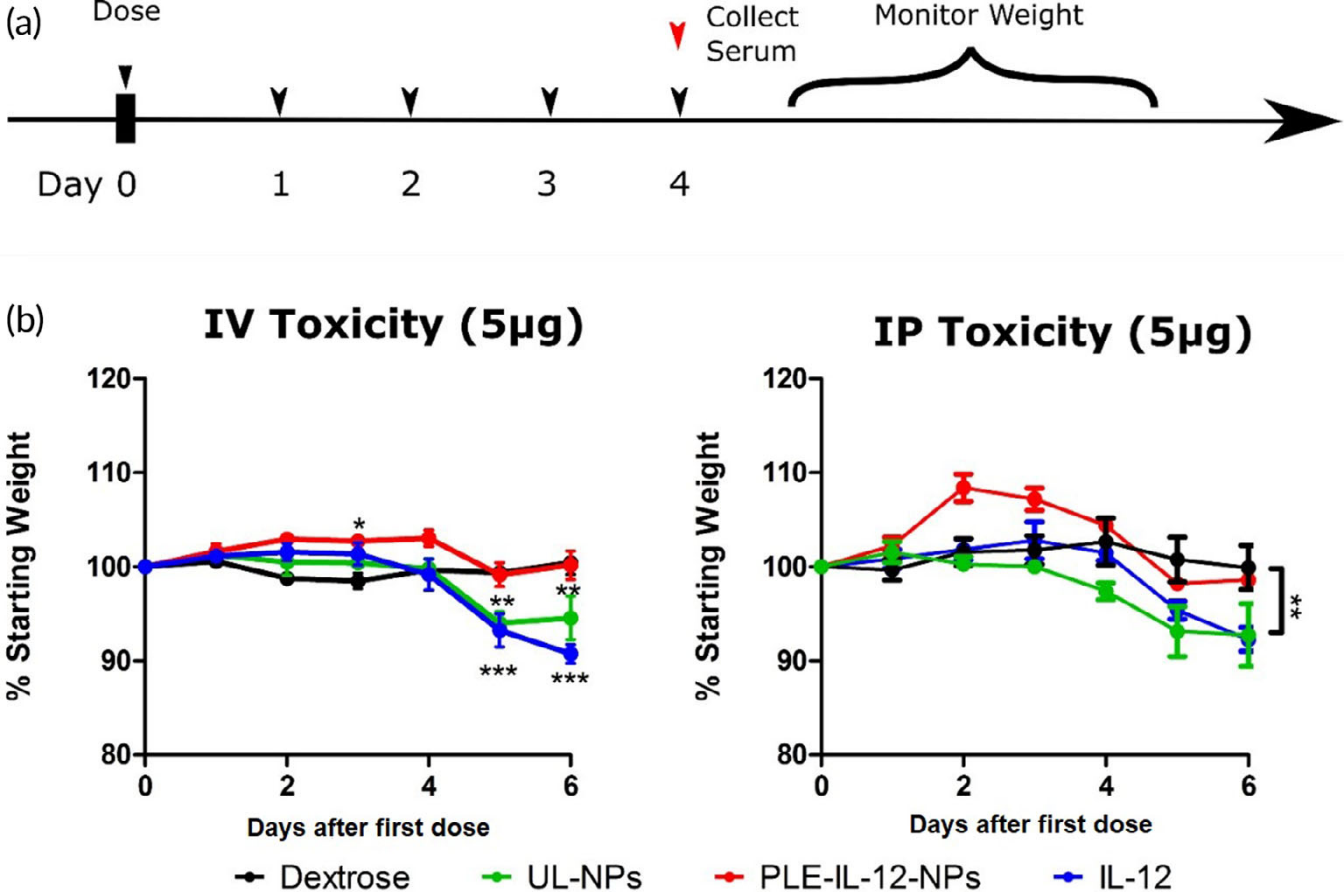
IL‐12 toxicity in healthy mice. (a), Schematic of dosing scheme in healthy animals. Mice were dosed with 5 μg IL‐12 in PLE‐IL‐12‐NPs, UL‐NPs, or carrier free and compared to 5% dextrose control. (b), Toxicity of various IL‐12 delivery methods administered IV (left) or IP (right) as measured by weight loss during and after dosing.**indicate *p* < 0.01 ***indicate *p* < .01 as measured by two‐way ANOVA with bonferroni post hoc test across all group *N* = 5

Having demonstrated that PLE‐IL‐12‐NPs accumulate in tumors following systemic administration, we wanted to confirm the delivery of the IL‐12 payload. Based on previous data, the majority of IL‐12 is released from PLE‐IL‐12‐NPs between 8 and 24 h,^33^ which overlaps well with the timing of NP concentration in the tumors (Figure S1b,c). To test the biodistribution of IL‐12 payload, organs from Figures S1b, and S3 were homogenized and assayed for IL‐12 content by ELISA (Figure S4). Note that this recovered IL‐12 includes both the delivered IL‐12 and endogenously produced IL‐12 in response to therapy and thus compounds itself as IL‐12 signaling can drive further IL‐12 production.^37^ There was a trend toward greater amounts of IL‐12 in the tumors and less off‐target exposure in the liver from PLE‐IL‐12‐NPs as compared to UL‐NPs by the 24 h time point (Figure S4). This suggests, coupled with previously demonstrated release data,^33^ that PLE‐IL‐12‐NPs selectively deliver IL‐12 to tumors, while UL‐NPs lose the attached IL‐12 in circulation which can then traffic as carrier‐free IL‐12. This suggests that PLE‐IL‐12‐NPs can reduce systemic exposure to IL‐12 upon systemic delivery. IFN‐γ levels were also measured as an indication of IL‐12 activity (Figure S4b) and followed similar trends to NP distribution.

### 3.2 PLE‐IL‐12‐NPs reduce severe toxicity of IL‐12 upon systemic administration

Because the biodistribution of PLE‐IL‐12‐NPs demonstrated concentration of both NP and payload in tumors we hypothesized that they may also limit the cytokine‐related toxicities that have been the main limiting factor for IL‐12 in the clinic^13^, ^14^, ^15^ upon systemic delivery. To this end, toxicity studies in healthy mice were performed (Figure 2a). Subjects were dosed with 5 μg IL‐12 in PLE‐IL‐12‐NPs, UL‐NPs, as a free cytokine, or vehicle control either IV or IP and monitored for weight change in response to treatment (Figure 2b). IL‐12 delivery from both UL‐NPs and carrier‐free IL‐12 showed significant, severe toxicity from both IV and IP delivery, with subjects losing ~10% body weight during and immediately after treatment. Conversely, delivery from PLE‐IL‐12‐NPs showed no significant weight loss compared to controls. This demonstrates a substantially safer toxicity profile from PLE‐IL‐12‐NPs not only compared to carrier‐free IL‐12 but also compared to UL‐NPs. These data demonstrate the critical role that layering the NP plays in targeting, preventing systemic activity, and reducing the severe off‐target toxicity of IL‐12 (Figure 2). Indeed, PLE‐IL‐12‐NPs show no significant short or long term off target immune activation related toxicity at measured doses.

### 3.3 PLE‐IL‐12‐NPs expand the therapeutic window of IL‐12 delivered systemically

**FIGURE 3:**
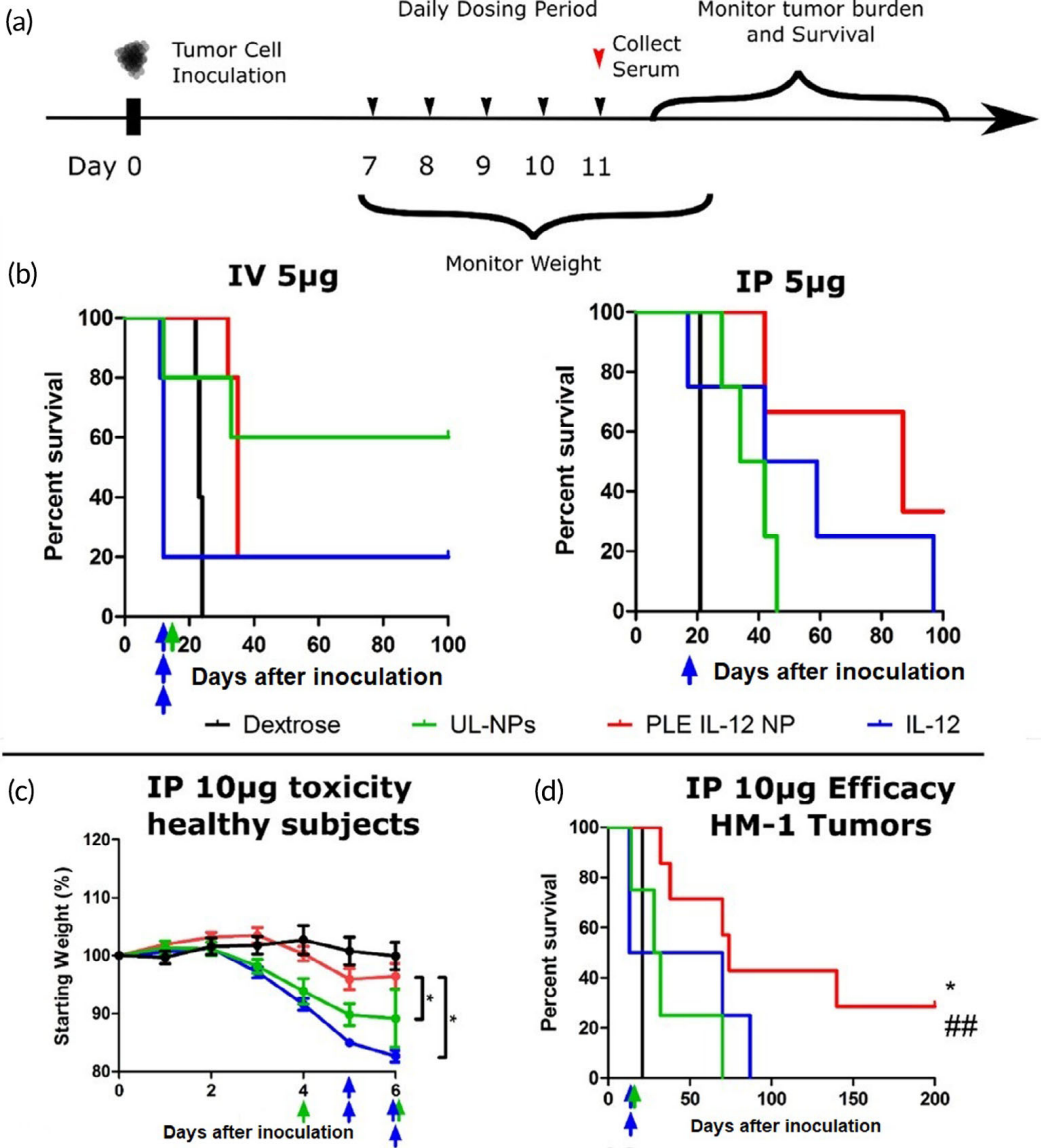
PLE‐IL‐12‐NPs improve anti‐tumor efficacy of IL‐12. (a) Schematic of dosing scheme in HM‐1 tumor bearing animals. Mice were dosed with 5 μg IL‐12 in PLE‐IL‐12‐NPs, UL‐NPs, or carrier free and compared to 5% dextrose control 7 days after IP inoculation of HM‐1 tumor cells. (b) Survival curves of 5 μg IL‐12 treated mice for IV (left, *n* = 5) and IP (right, *n* = 4 Dex, UL‐NPs, IL‐12; *N* = 3 PLE‐IL‐12‐NPs) delivery routes. Colored arrows indicate toxicity related deaths during and immediately following dosing. (c) Animal weights upon daily dosing of 10 μg of IL‐12 for five doses from PLE‐IL‐12‐NPs (red), UL‐NPs (green), or carrier‐free (blue) compared to vehicle control (black). *N* = 5. (d) Survival of mice with orthotopic HM‐1 tumors treated 7 days after inoculation with five daily doses of IL‐12 from different vehicles similar to (a). *N* = 4 for PBS, IL‐12, and UL‐NPs, *n* = 7 for PLE‐IL‐12‐NPs *indicates *p* < 0.05 compared to UL‐NPs ## indicates *p* < 0.01 compared to Dextrose as measured by Mantel–Cox test between indicated groups

Given the enhanced concentration of IL‐12 in tumors and a subsequent reduction in severe toxicity mediated by PLE‐IL‐12‐NPs we next tested the NPs anti‐tumor efficacy. Mice were inoculated with orthotopic HM‐1 tumors that were allowed to form for 7 days prior to 5 daily treatments with 5 μg of IL‐12 given carrier‐free, from PLE‐IL‐12‐NPs, or from UL‐NPs (Figure 3a) and compared to controls. Subjects were monitored for severe toxicity during and immediately after dosing by weight changes (Figure S5a,b). These data demonstrate that the PLE‐IL‐12‐NP treated animals were healthier than the carrier‐free IL‐12, though due to the confounding variable of the presence of tumors and ascites, toxicity measured by weight loss of free IL‐12 was more muted than in healthy mice (Figure 2). However, tumor burden as measured by fluorescence signal on IVIS (Figure S5c,d) and survival (Figure 3b) showed that PLE‐IL‐12‐NP given IP generated more robust anti‐tumor responses than UL‐NPs or free‐IL‐12 and led to long‐term survival of one out of three mice.

In contrast to IP delivery, IV delivery showed a more muted antitumor response with PLE‐IL‐12‐NPs, while UL‐NPs showed an enhanced response. This reduced response of PLE‐IL‐12‐NPs could be because the tumors in these studies were relatively small and not fully vascularized. UL‐NPs release IL‐12 more readily in the blood stream over extended times than PLE‐IL‐12‐NPs as evidenced by the toxicity data (Figure 2) which likely allows for easier access of this released IL‐12 as a free cytokine to the tumors as compared to much larger layered NPs that cannot extravasate readily into the tumor tissue. However, the UL‐NP delivered IV is also a main route of severe toxicity with one out of four tumor bearing mice treated with UL‐NPs succumbing to toxic side effects—thus this approach is not a viable treatment.

A further test was carried out at twice the previous dose of IL‐12 to test the limits of PLE‐IL‐12‐NP mediated toxicity when given IP. Significant toxicities occurred at this increased dosing levels in both the carrier‐free IL‐12 and UL‐NP treated subjects regardless of tumor status. In healthy animals, all mice treated with carrier‐free IL‐12 and 50% of the mice treated with UL‐NPs needed to be sacrificed during or immediately after dosing due to severe toxicity as measured by severe (>15%) body weight reduction (Figure 3c). In tumor‐bearing mice, both carrier‐free and UL‐NP IL‐12 treatments showed significant severe toxicity (Figure S5e), with multiple subjects succumbing to toxicity during the dosing period. In contrast, regardless of tumor burden, PLE‐IL‐12‐NPs were well tolerated, inducing minimal changes in body weight and condition (Figure 3c), and causing zero toxicity‐related deaths (Figure 3d). Furthermore, PLE‐IL‐12‐NPs prolonged survival (Figure 3d) compared to other IL‐12 delivery methods in these studies and led to cure in 2 out of 7 treated mice.

**FIGURE 4:**
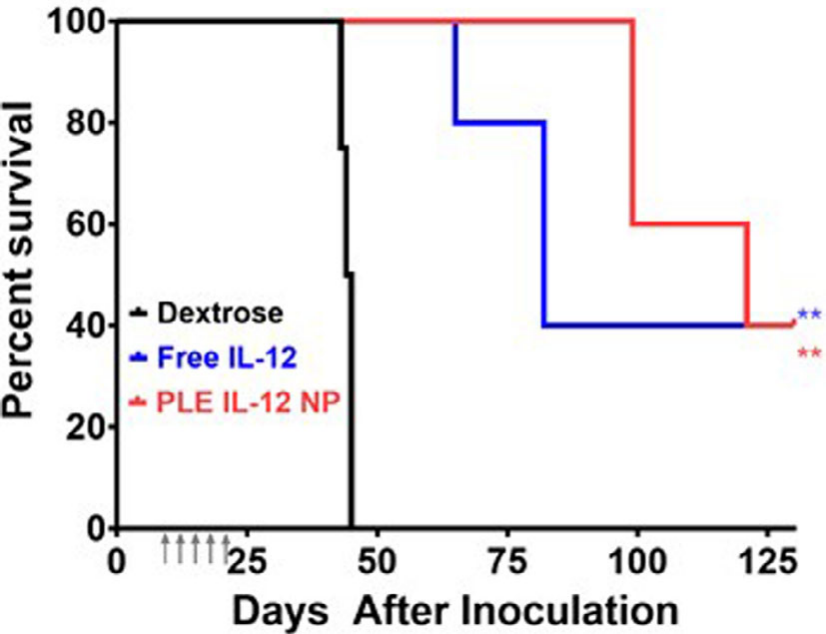
Survival of mice bearing orthotopic ovarian KPCA tumors (1E06 IP). Tumors were allowed to establish for 7 days prior to beginning five daily treatments at 10 μg equivalent of IL‐12 (gray arrows). **indicates a significant increase in median survival relative to dextrose controls evaluated using the Log‐Rank test (*p* < 0.01). *N* = 4 Dex; *N* = 5 IL‐12, NPs

These results show promise for safely driving an effective antitumor immune response in refractory ovarian tumors. However, many tumors can present differently and as such we used a second ovarian tumor model that presents with a more “cold” tumor environment to demonstrate the ability of PLE‐IL‐12‐NPs to safely drive an effective antitumor response even in the most refractory tumors. Similar degrees of efficacy were obtained in a genetically distinct cold model of ovarian cancer (KPCA, with clinically relevant mutations in KRAS^G12V^TrP53^R172H^Ccne1^OE^Akt2^OE^) also implanted orthotopically and treated identically (Figure 4). Importantly, the KPCA model was previously shown to be an immunologically cold tumor (low T‐cell infiltration, higher proportions of T_Regs_, and large fractions of suppressive myeloid cells) that required an aggressive treatment strategy of the CHK1 inhibitor Prexasertib alongside CTLA4 and PD‐L1 inhibition to reach long‐term cures. In contrast, PLE‐IL‐12 NPs as a single‐agent therapy achieved long‐term survival in two out of five mice. These data demonstrate the importance of driving an immune response in these cold tumors for effective treatment, which requires an effective delivery vehicle to do so safely.

### 3.4 PLE‐IL‐12‐NPs enhance immune activity in tumors upon systemic delivery

**FIGURE 5:**
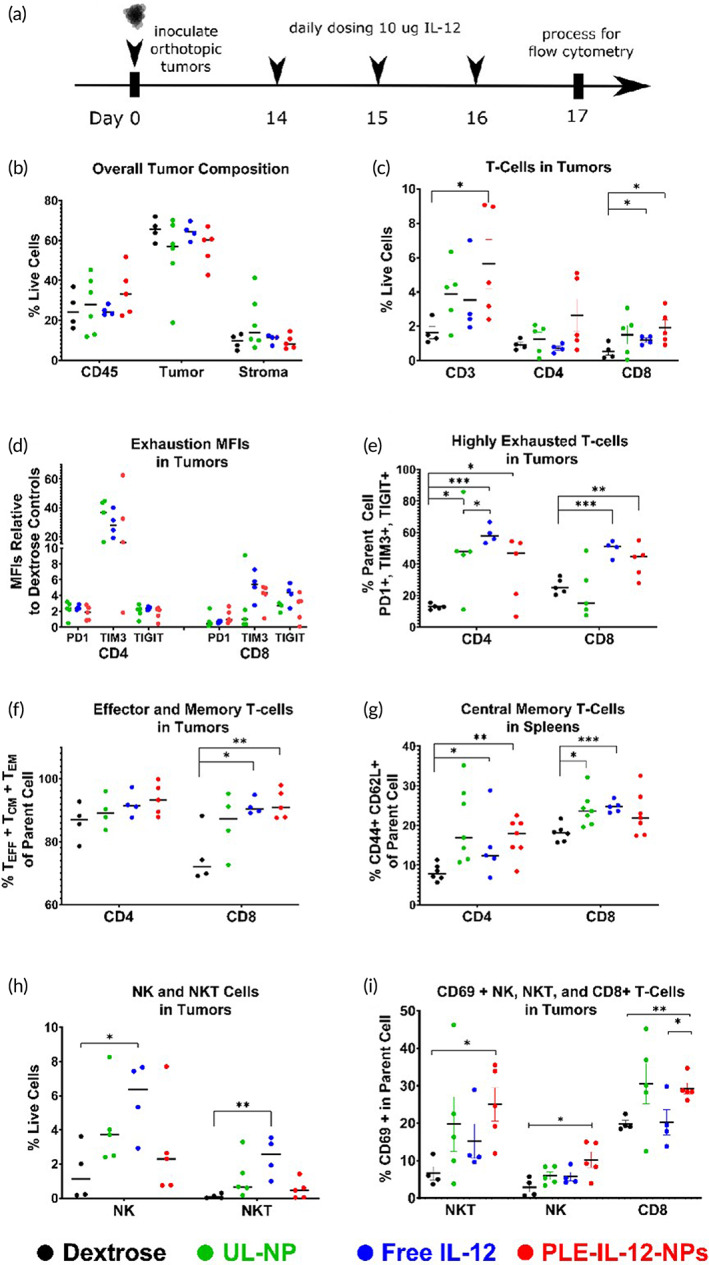
PLE‐IL‐12‐NPs show an equivalent immune response to carrier‐free IL‐12. (a) Experimental design for immune profiling. Mice were inoculated with orthotopic HM‐1 tumors and dosed with 10 μg IL‐12 IP in PLE‐IL‐12‐NPs, UL‐NPs, or carrier‐free and compared to vehicle control for 3 daily doses 14 days after inoculation. (b,c) Immune population statistics found within the tumor environment as measured by flow cytometry. (d,e) Exhaustion indicators found in the tumor environment. (f,g) Memory response found in the tumor and spleen by flow cytometry. (h,i) Effector responses as measured by flow cytometry. Statistical differences were measured by the Student's *t* test. *p < 0.05; **p < 0.01; ***p < 0.001. *N* = 6 (Dextrose, ULNP, LNP), *N* = 5 (Free IL‐12) unless reduced due to insufficient events during analysis

**FIGURE 6:**
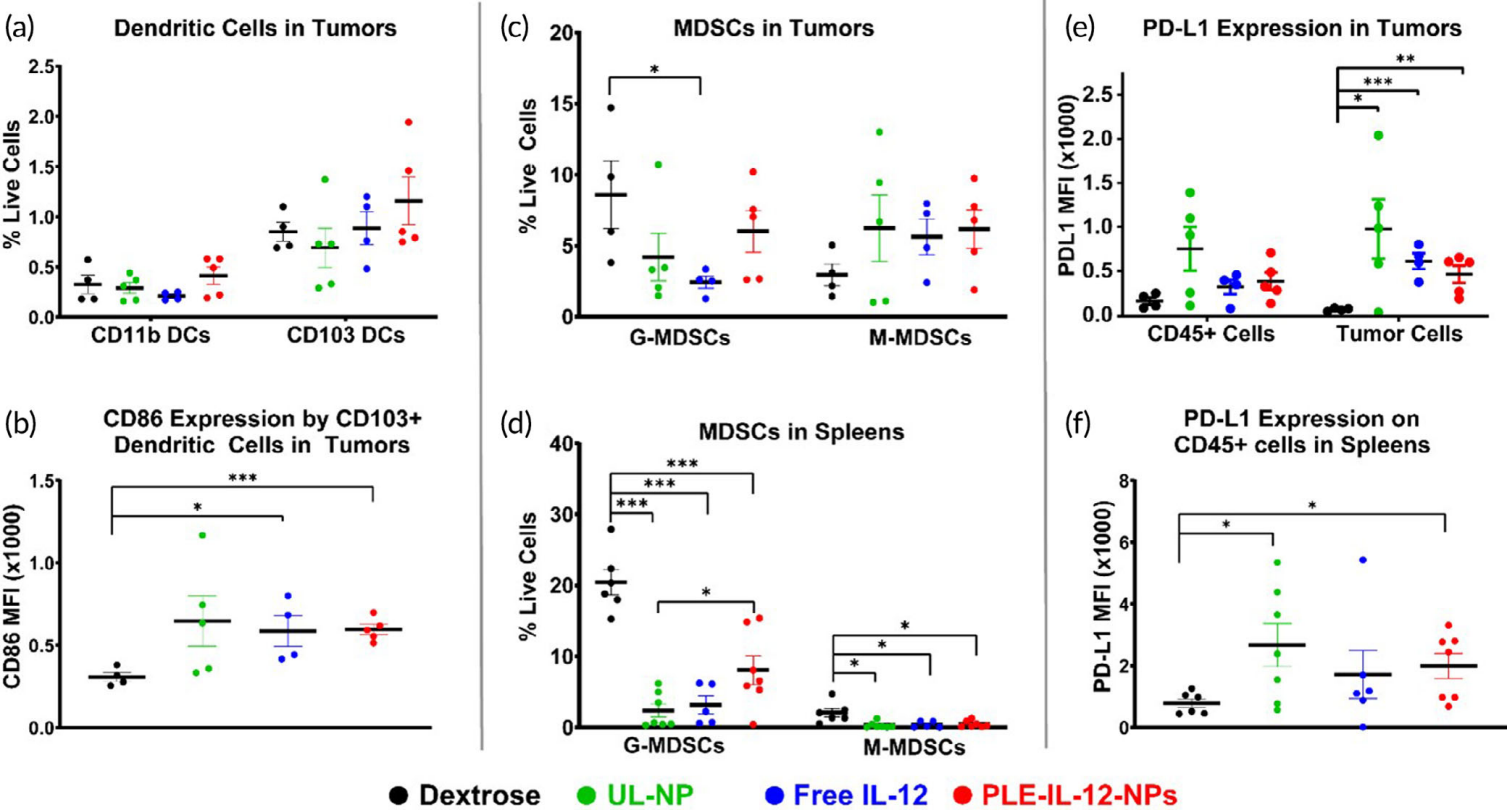
PLE‐IL‐12‐NPs engage myeloid cells and checkpoint inhibition in the tumor and spleens. HM‐1 tumors were allowed to establish for 14 days before dosing with 10 μg IL‐12 IP in PLE‐IL‐12‐NPs, UL‐NPs, or carrier‐free and compared to vehicle control. Tissues were harvested for analysis 24 h after the third daily dose. (a–f) Immune populations found within the tumor environment (a,b,c,e) and spleen (d,f) as measured by flow cytometry. Statistical differences were measured by the Student's *t* test. *p < 0.05; **p < 0.01; ***p < 0.001. *N* = 6 (Dextrose, ULNP, LNP), *N* = 5 (Free IL‐12) unless reduced due to insufficient events during analysis

We next analyzed the immunological response triggered by PLE‐IL12‐NPs using flow cytometry. HM‐1 tumors were established for 14 days after IP implantation before treating with three daily doses of 10 μg IL‐12 equivalent from PLE‐IL‐12‐NPs, UL‐NPs, carrier‐free IL‐12 or dextrose control (Figures 5 and 6). Ascites, tumors, and spleens were harvested 24 h after the final treatment. Samples were profiled for diverse T‐cell phenotypes including CD4/8 subtypes, activity markers, effector/memory markers, and exhaustion markers. Myeloid populations were also assessed including macrophages, dendritic cells (DCs), and myeloid‐derived suppressor cells (MDSCs) (gating strategy Figures S6 and S7, complete overview by tissue Figure S8).

There were no differences in the number of total immune (CD45+) cells within tumors after treatment (Figure 5b), but there were changes in key phenotypes. There was a shift toward enhanced T cell infiltration from all three tested therapies, with the greatest tumor infiltration of CD3+ and CD8+ T‐cells caused by PLE‐IL‐12‐NPs (Figure 5c). Further investigation of the exhaustion phenotypes of those T‐cells revealed that all three IL‐12 therapies increased expression of PD‐1, TIGIT, and TIM3 (Figure 5d) in both CD4+ and CD8+ T‐cells. Strikingly, TIM3 expression was upregulated by 20–40 fold in CD4+ and 4–6 fold in CD8+ T‐cells for all three therapies compared to dextrose treated tumors. TIM3 is known the be transiently upregulated by IFN‐γ‐expressing activated T‐cells with its inhibitory properties becoming clearer after extended expression.^38^ Nonetheless, this may suggest TIM3 blockade as a potential candidate for combination therapy with IL‐12 in these tumors. All three IL‐12 treatments increased the fraction of CD4+ T‐cells that were highly exhausted (PD‐1, TIM3, and TIGIT triple positive) while free IL‐12 and PLE‐IL‐12‐NPs but not UL‐NPs lead to an increase in the fraction of highly exhausted CD8+ T‐cells. PLE‐IL‐12‐NPs and free IL‐12 also shifted the CD8+ T‐cells toward effector and/or memory subtypes within the tumors (Figure 5f) and a CD4+ central memory phenotype in the spleen (Figure 5g).

Infiltration of NK and NKT cells was only increased upon free IL‐12 administration (Figure 5g). However, PLE‐IL‐12‐NPs elicited the highest degree of NK, NKT, and CD8+ T‐cell activation indicated by CD69 expression (Figure 5h). Taken together, this data suggests that PLE‐IL‐12 NPs are at least as effective as free IL‐12 at driving cytotoxic T‐cell infiltration into tumors, enhancing a memory response locally and systemically, and are more effective than UL‐NPs or free IL‐12 at activating innate and adaptive cytolytic cells in the tumors.

PLE‐IL‐12‐NPs also impacted antigen presenting cells and myeloid populations locally and systemically while increasing the presentation of checkpoint molecules (Figure 6). While the frequency of infiltrating antigen presenting cells in the tumor was not significantly impacted by the therapies (Figure 6a), the costimulatory molecule CD86 was upregulated in CD103+ DCs after PLE‐IL‐12‐NPs or free IL‐12 therapy (Figure 6b), suggesting an enhanced activation of those cells. Interestingly, only free IL‐12 decreased the amount of MDSCs in the tumor (3‐fold reduction, Figure 6c), but all three therapies decreased MDSCs in the spleen (Figure 6d) with PLE‐IL‐12‐NPs providing less reduction (2‐fold G‐MDSCs and 4‐fold M‐MDSCs) than free IL‐12 (6‐ and 5‐fold) or UL‐NPs (10‐ and 5‐fold). Finally, PD‐L1 expression was upregulated in the tumor (~4× over dextrose control) but not the CD45+ immune cells (Figure 6e) for all three treatments. This, coupled with a similar level of PD‐L1 expression in the spleen for all three treatments (Figure 6f), suggests a robust expression of IFN‐γ (a known inducer of PD‐L1 expression) at the tumor site.

## 4 DISCUSSION

In this work we demonstrate that the rational engineering of a NP delivery vehicle using the LbL technique makes significant improvements to systemic IL‐12 therapy, not only compared to carrier‐free cytokine delivery but also in comparison with a simpler NP design (UL‐NPs) that does not incorporate any rational engineering of NP properties. Another critical finding in this work is the pronounced efficacy and immune activation in ovarian tumors, which have been refractory to many other forms of immunological treatments, opening the door to combination treatments and additional improvements in immunotherapy to this previously unresponsive malignancy. We demonstrate the ability of PLE‐IL‐12‐NPs to drive immune infiltration into the cold tumor microenvironment of high‐grade ovarian cancer with great efficacy without inducing the severe toxicity known for with IL‐12 treatment. The ability to initiate a robust anti‐tumor response in an orthotopic metastatic ovarian cancer is a significant indication of promise for translational therapies.

Perhaps the most critical finding in this study is the demonstration of not only reduced toxicity but also increased efficacy of IL‐12 therapy through a systemic delivery vehicle. The main impediment to clinical application of IL‐12 has always been its associated toxicities. To this end, any future IL‐12 studies must demonstrate reduction in systemic exposure while maintaining activity in the tumor. Many recent delivery techniques such as microparticles and hydrogels achieve this result by limiting systemic leakage from tumors. However, these strategies are limited to local injection of tumors directly, which is not possible in many epithelial tumors, including ovarian cancers, which do not have a singular main tumor mass to inject or easily accessible tumors for multiple injections. The described PLE‐IL‐12‐NPs are not limited to local or intratumoral treatments. Our data demonstrate a robust IL‐12 pathway immune response against ovarian cancer with PLE‐IL‐12‐NP treatment. The therapeutic response from PLE‐IL‐12‐NPs showed greater efficacy compared to both free cytokine and UL‐NPs. Immunologically, PLE‐IL‐12‐NPs drove only modest changes over the other IL‐12 therapies, but the layered particles were able to promote a number of statistically different phenotypes that were not achieved with unlayered particles including an increase in T‐Cells, CD8+ T‐Cells, effector memory T‐Cells, highly exhausted CD8+ T‐Cells, and CD69 upregulation on T‐cells and NK cells in the tumors. However, more important is the demonstration that these immune responses are achieved with PLE‐IL‐12‐NPs at a greatly improved toxicity level compared to other delivery techniques.

Another key finding in the reported work is the enhancement of IL‐12 therapy from PLE‐IL‐12‐NPs over UL‐NPs. We demonstrate throughout that the engineered LbL NP structure is critical to the reduction of severe toxicity as well as the enhancement of efficacy in these experiments, as a simpler unmodified liposomal particle does not show the same results in both toxicity and efficacy. The LbL particles were designed with thorough consideration of the design challenges required for successful cytokine therapy including efficient protein encapsulation, proficient maintenance of cytokine activity from particles, maintenance of cytokine access to surface receptors within the tumor environment, and selective interaction with tumor cells to concentrate both NP and payload in the tumor to achieve active levels of signaling in the tumor, a critical consideration for IL‐12 success,^39^ while preventing off target activity which leads to toxicity.

Perhaps most importantly, in this work we demonstrate that PLE‐IL‐12‐NPs are capable of pronounced single‐agent efficacy in ovarian tumors driving robust infiltration of anti‐tumor immune cells into relatively “cold” tumors. This is a critical finding in ovarian tumors as they have been refractory to most immunotherapy strategies to date. Toxicity concerns in ovarian cancer patients are typically elevated by severe comorbidities, often limiting the application of checkpoint inhibitors, much less proinflammatory cytokines. Herein we demonstrate that PLE‐IL‐12‐NPs are capable of a nontoxic increase in proinflammatory immune activity in ovarian cancer (Figure 3) and this increase is maintained even in a demonstrated “cold” tumor environment (Figure 4). Indeed, we show that this increased proinflammatory immune response within the tumor is capable of a pronounced single‐agent response in these previously refractory tumors. However, there is potential for further success in these difficult to treat tumors through combination with checkpoint inhibition or other immunotherapies, which has been deemed a promising path forward for improving immune outcomes.^40^, ^41^, ^42^ As IL‐12 delivery drives the proinflammatory immune response within the tumor, so too is the exhaustion of T cells driven, as demonstrated herein (Figure 5). By adding a checkpoint inhibitor such as an antibody against PD‐L1^43^ or TIM3, both of which showed marked increases after IL‐12 therapy, a further improvement in anti‐tumor response is conceivable. Beyond combination with checkpoint inhibitors, combination therapy with additional cytokines is also likely to improve this therapy.^42^ Finally, in this study we focus on IL‐12 delivery from the described LbL‐NPs; however, this design is modular and could easily enable the delivery of other synergistic cytokines as well as combinations of cytokines within the same NP construct.

## AUTHOR CONTRIBUTIONS

**Antonio E. Barberio:** Conceptualization (lead); data curation (lead); formal analysis (lead); funding acquisition (supporting); investigation (lead); methodology (lead); project administration (equal); validation (equal); visualization (lead); writing – original draft (lead); writing – review and editing (equal). **Sean G. Smith:** Conceptualization (lead); data curation (lead); formal analysis (lead); funding acquisition (supporting); investigation (lead); methodology (lead); project administration (equal); validation (equal); visualization (lead); writing – original draft (lead); writing – review and editing (equal). **Ivan S. Pires:** Data curation (equal); formal analysis (supporting); investigation (supporting); methodology (supporting); project administration (supporting); writing – review and editing (equal). **Sonia Iyer:** Conceptualization (supporting); data curation (equal); formal analysis (equal); investigation (equal); methodology (equal); project administration (equal); validation (equal); visualization (supporting); writing – review and editing (equal). **Ferenc Reinhardt:** Data curation (equal); methodology (equal); writing – review and editing (equal). **Mariane B. Melo:** Conceptualization (supporting); data curation (equal); formal analysis (supporting); investigation (supporting); methodology (equal); project administration (equal); writing – review and editing (equal). **Heikyung Suh:** Data curation (equal); resources (equal); writing – review and editing (equal). **Robert A. Weinberg:** Conceptualization (supporting); formal analysis (supporting); funding acquisition (equal); investigation (supporting); methodology (supporting); project administration (supporting); resources (equal); validation (supporting); writing – review and editing (equal). **Darrel J. Irvine:** Conceptualization (equal); formal analysis (supporting); funding acquisition (lead); investigation (equal); methodology (equal); project administration (equal); resources (equal); validation (equal); writing – review and editing (equal). **Paula T. Hammond:** Conceptualization (equal); data curation (supporting); formal analysis (equal); funding acquisition (lead); investigation (equal); methodology (supporting); project administration (lead); resources (lead); supervision (lead); validation (lead); visualization (equal); writing – review and editing (lead).

## FUNDING INFORMATION

This work was supported by the National Cancer Institute (NCI, 1‐R01‐CA235375) and the Koch Institute Marble Center for Cancer Nanomedicine. Additional support is from the Marble Center for Cancer Nanomedicine Fellowship (SGS), NIH interdepartmental biotechnology training program (AEB) and an NCI F32 CA247210‐01A1 (SGS). Research facilities were supported in part by the Koch Institute Support Grant (P30‐CA14051) from the NCI and the MIT MRSEC Shared Experimental Facilities Grant (DMR‐0819762) from the National Science Foundation. S. Iyer was supported by the postdoctoral fellowship by Ludwig Fund for Cancer Research and Amgen. R.A. Weinberg was funded by grants from the NIH (R01 CA0784561 and P01 CA080111), Samuel Waxman Cancer Research Foundation, Breast Cancer Research Foundation, and Ludwig Fund for Cancer Research.

## CONFLICT OF INTEREST

PTH holds patents on the nanoparticle platform (patent no. 10278927, Layer‐by‐Layer Based Nanoparticles for Systemic Delivery Applications); these technologies are currently under development through separate funding from Shepherd Pharmaceutical and Novartis for targeted delivery applications toward cancer therapies and immunotherapies, separate from the work described here.

### PEER REVIEW

The peer review history for this article is available at https://publons.com/publon/10.1002/btm2.10453.

## Supporting information


**Figure S1.** Biodistribution of NPs upon systemic delivery. (a) Schematic of biodistribution study design. (b) Representative fluorescence results as measured by IVIS in tumors following 4‐h injection of PLE‐IL‐12‐NPs and UL‐NPs by both IV and IP delivery route. (c) Mean percent recovered fluorescence normalized by tissue weight (UL‐NPs, PLE‐IL‐12‐NP 24 h IV *n* = 3; 4 and 24 h IP PLE‐IL‐12‐NPs *n* = 5, error bars denote SEM). IVIS measurements from (b) were normalized by dextrose control treated subjects and percent recovered fluorescence was calculated with respect to all measured organs (liver, kidney, spleen, and tumor). *indicates *p* < 0.05 as calculated by one‐tailed *t* test. (d) Correlation of % recovered IL‐12/g tissue to % recovered fluorescence/g tissue for both IV (left) and IP (right) delivery within tumors. Linear regression performed using GraphPad PRISM showing 95% confidence ranges and slopes. ****indicates *p* < 0.0001 for slope differing from zero.

**Figure S2.** Sample Images from IVIS BioD studies of all collected organs.

**Figure S3.** Biodistribution in the kidney and spleen as measured by fluorescence.

**Figure S4.** IL‐12 and IFN‐γ recovery upon systemic IL‐12 delivery. Results are corrected for baseline values from untreated subjects. (not significant).

**Figure S5.** (a,b) 5 μg dosed IL‐12 tumor‐bearing mice toxicity (c,d) 5 μg dosed IL‐12 tumor burden E 10 μg IP dosed IL‐12 tumor‐bearing mice toxicity. Arrows indicate toxicity induced deaths.

**Figure S6:** T‐cell gating strategies for memory (upper) and exhaustion phenotypes (lower).

**Figure S7:** Gating strategy for myeloid cells in the tumor and ascites (top) and spleen (bottom).

**Figure S8:** Overall changes in immune milieu for each tissue. Statistical differences were measured by the Student's *t*‐test with respect to dextrose controls. *p < 0.05; **p < 0.01; ***p < 0.001.

## Data Availability

The data for this study are available within the article, with additional data available in the Supporting Information.
