# Supplementary figures and images for: Layer‐by‐layer interleukin‐12 nanoparticles drive a safe and effective response in ovarian tumors
Source: Bioeng Transl Med. 2022 Dec 1;8(2):e10453. doi: 10.1002/btm2.10453 (PMC10013828; doi:10.1002/btm2.10453)

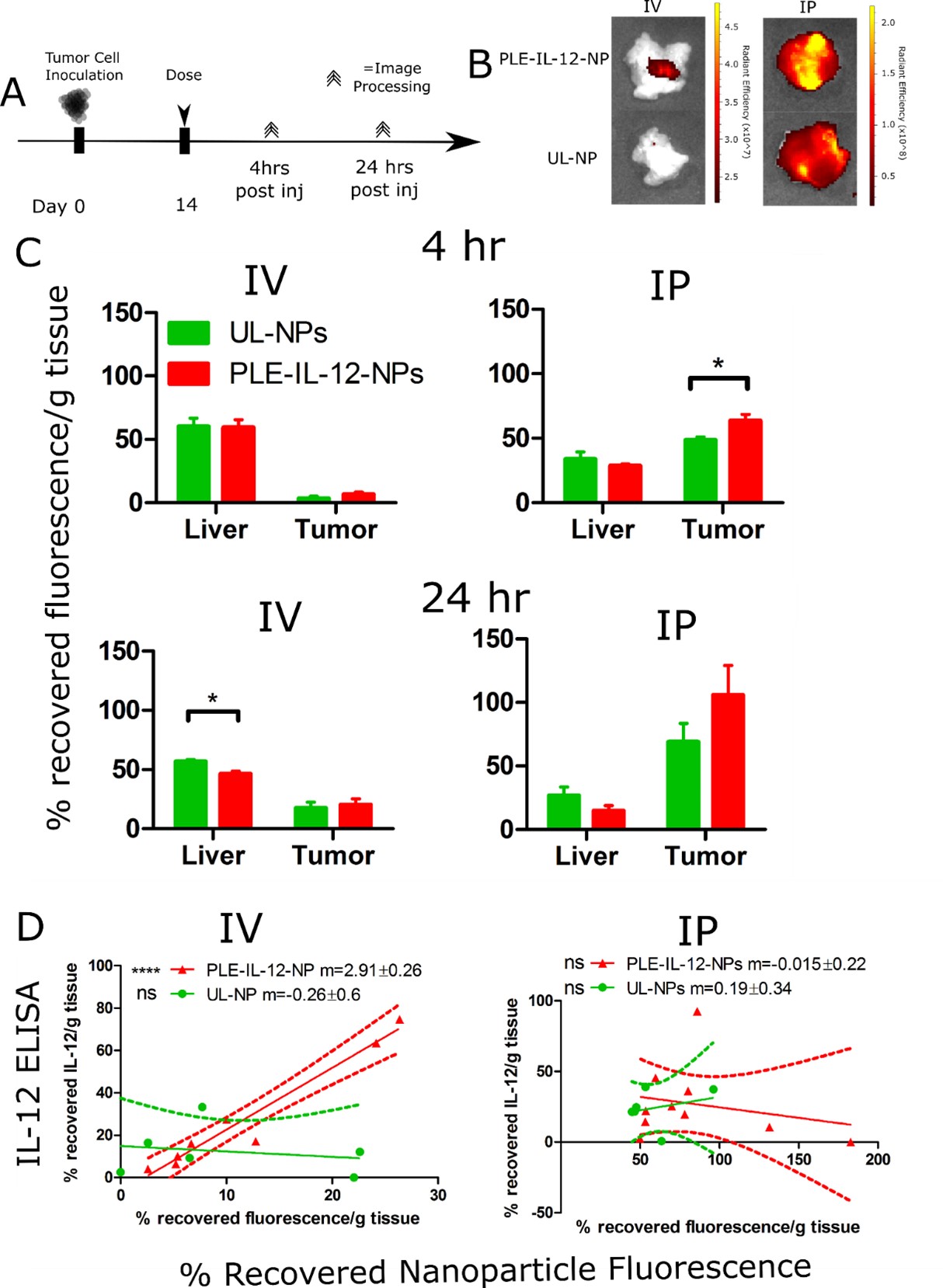

Supplement: Supplementary file 1 — Figure S1. Biodistribution of NPs upon systemic delivery. (a) Schematic of biodistribution study design. (b) Representative fluorescence results as measured by IVIS in tumors following 4‐h injection of PLE‐IL‐12‐NPs and UL‐NPs by both IV and IP delivery route. (c) Mean percent recovered fluorescence normalized by tissue weight (UL‐NPs, PLE‐IL‐12‐NP 24 h IV n = 3; 4 and 24 h IP PLE‐IL‐12‐NPs n = 5, error bars denote SEM). IVIS measurements from (b) were normalized by dextrose control treated subjects and percent recovered fluorescence was calculated with respect to all measured organs (liver, kidney, spleen, and tumor). *indicates p < 0.05 as calculated by one‐tailed t test. (d) Correlation of % recovered IL‐12/g tissue to % recovered fluorescence/g tissue for both IV (left) and IP (right) delivery within tumors. Linear regression performed using GraphPad PRISM showing 95% confidence ranges and slopes. ****indicates p < 0.0001 for slope differing from zero. [file BTM2-8-e10453-s007.jpg]

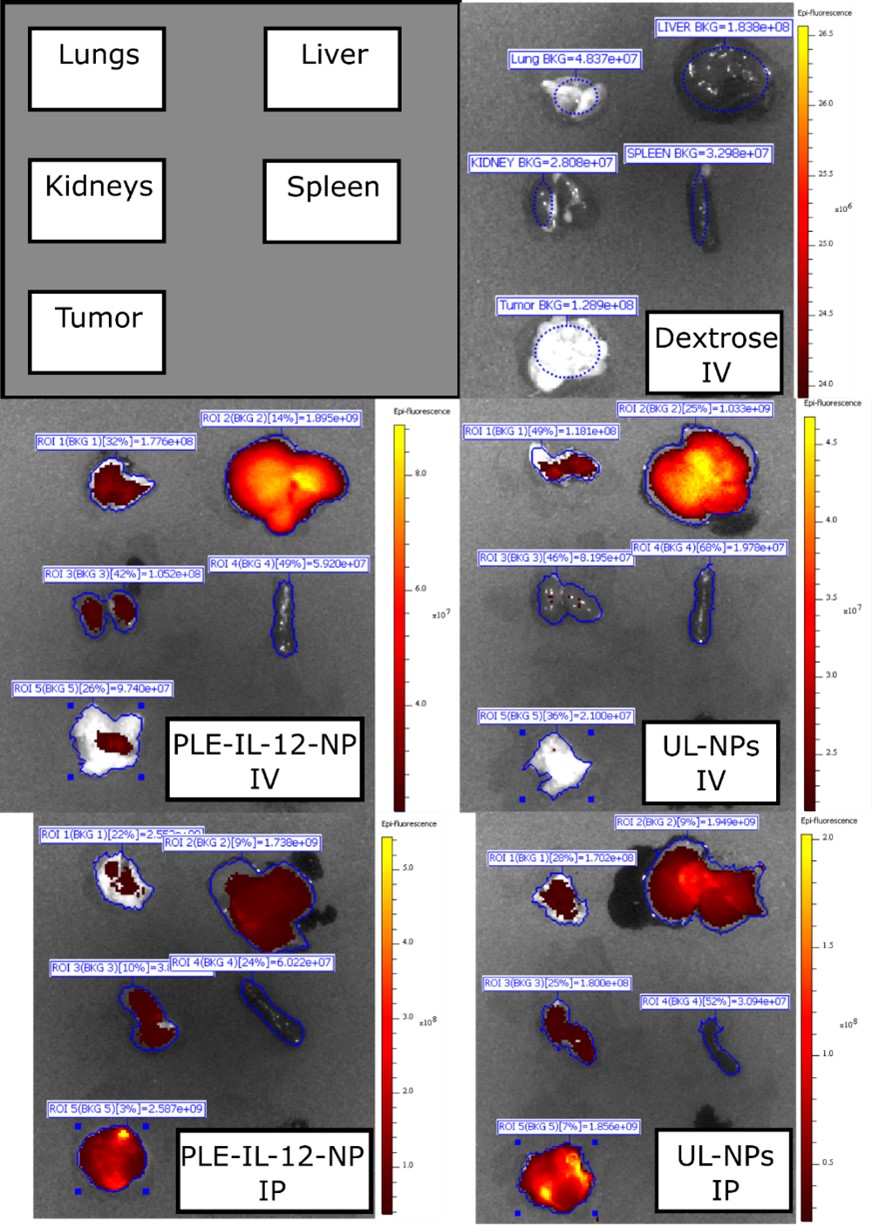

Supplement: Supplementary file 2 — Figure S2. Sample Images from IVIS BioD studies of all collected organs. [file BTM2-8-e10453-s001.jpg]

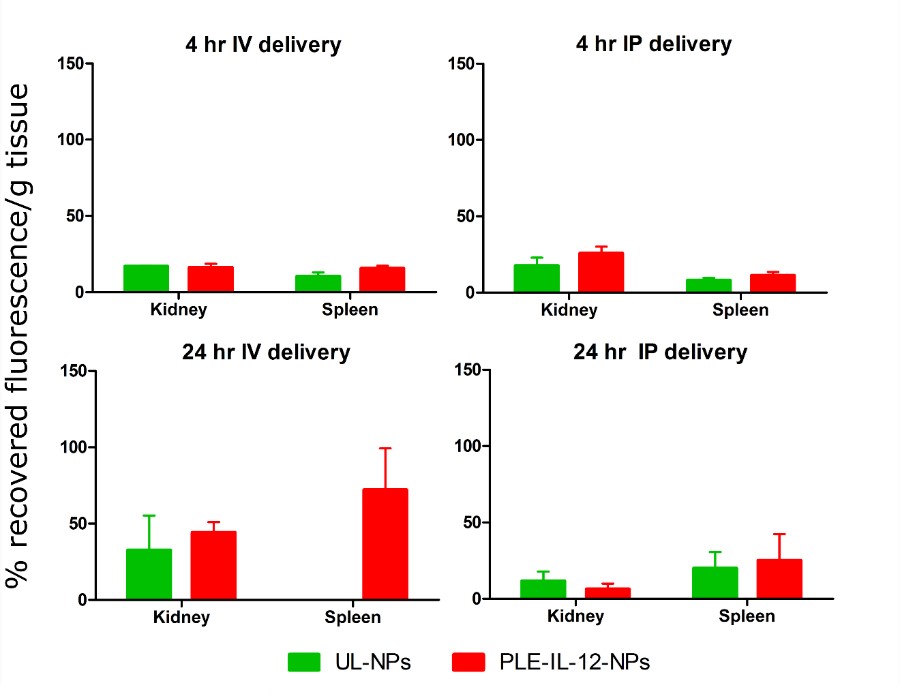

Supplement: Supplementary file 3 — Figure S3. Biodistribution in the kidney and spleen as measured by fluorescence. [file BTM2-8-e10453-s008.jpg]

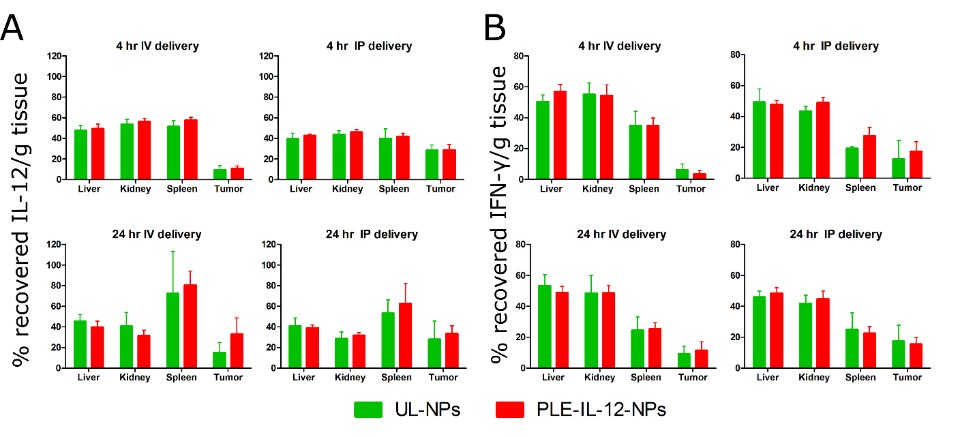

Supplement: Supplementary file 4 — Figure S4. IL‐12 and IFN‐γ recovery upon systemic IL‐12 delivery. Results are corrected for baseline values from untreated subjects. (not significant). [file BTM2-8-e10453-s004.jpg]

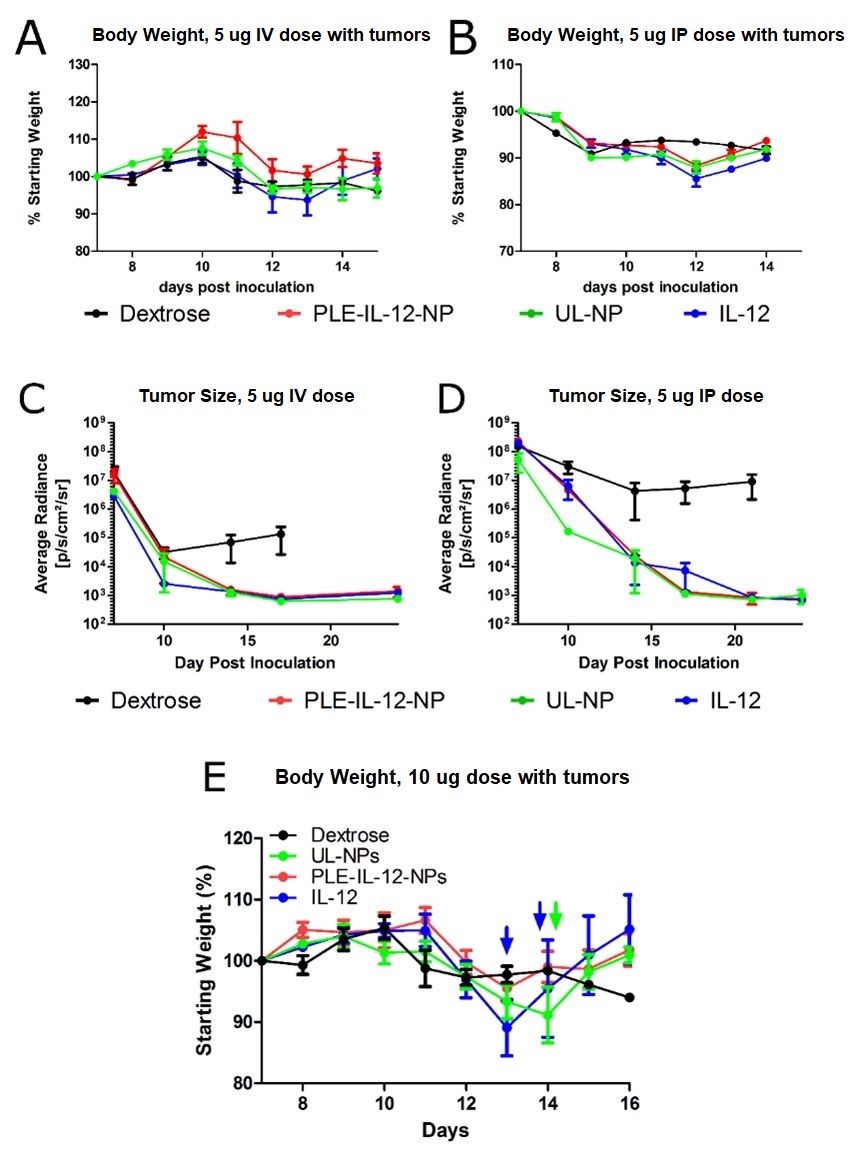

Supplement: Supplementary file 5 — Figure S5. (a,b) 5 μg dosed IL‐12 tumor‐bearing mice toxicity (c,d) 5 μg dosed IL‐12 tumor burden E 10 μg IP dosed IL‐12 tumor‐bearing mice toxicity. Arrows indicate toxicity induced deaths. [file BTM2-8-e10453-s003.jpg]

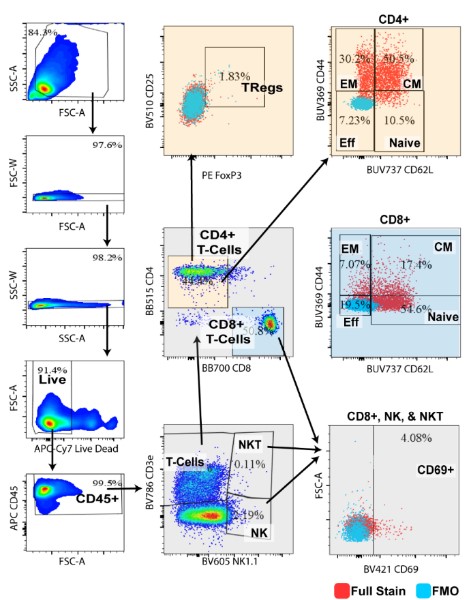

Supplement: Supplementary file 6 — Figure S6: T‐cell gating strategies for memory (upper) and exhaustion phenotypes (lower). [file BTM2-8-e10453-s002.zip › btm210453-sup-0006-FigureS6.jpg]

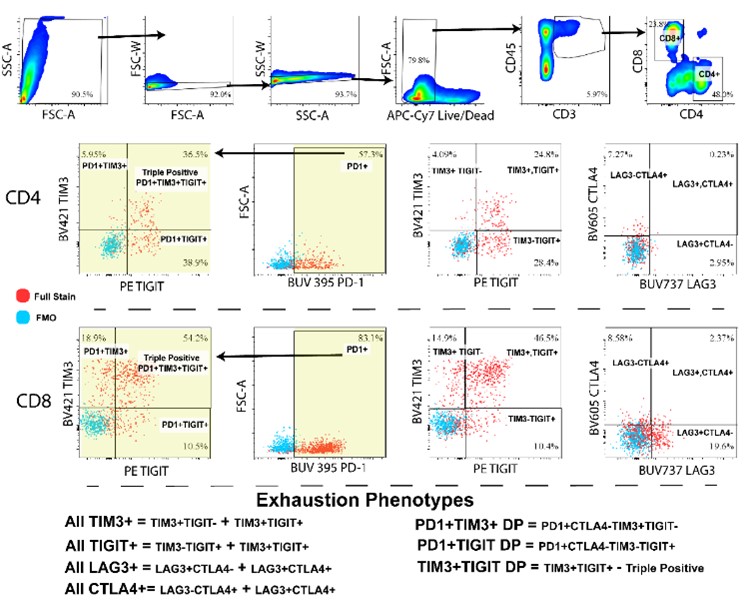

Supplement: Supplementary file 6 — Figure S6: T‐cell gating strategies for memory (upper) and exhaustion phenotypes (lower). [file BTM2-8-e10453-s002.zip › btm210453-sup-0007-Figure S6 P2.jpg]

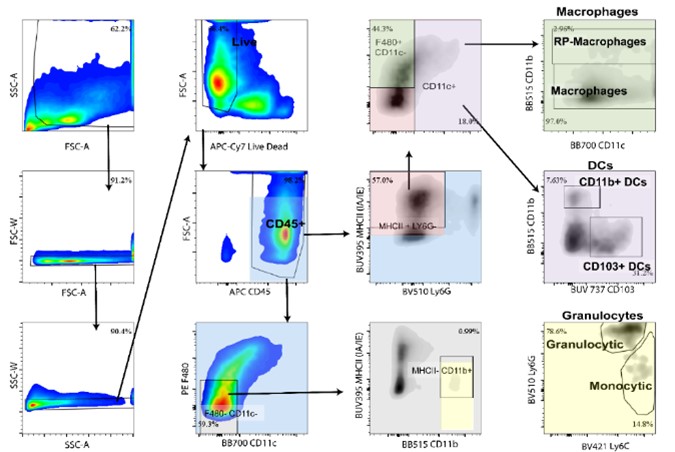

Supplement: Supplementary file 7 — Figure S7: Gating strategy for myeloid cells in the tumor and ascites (top) and spleen (bottom). [file BTM2-8-e10453-s005.zip › btm210453-sup-0008-Figure S7 bottom.jpg]

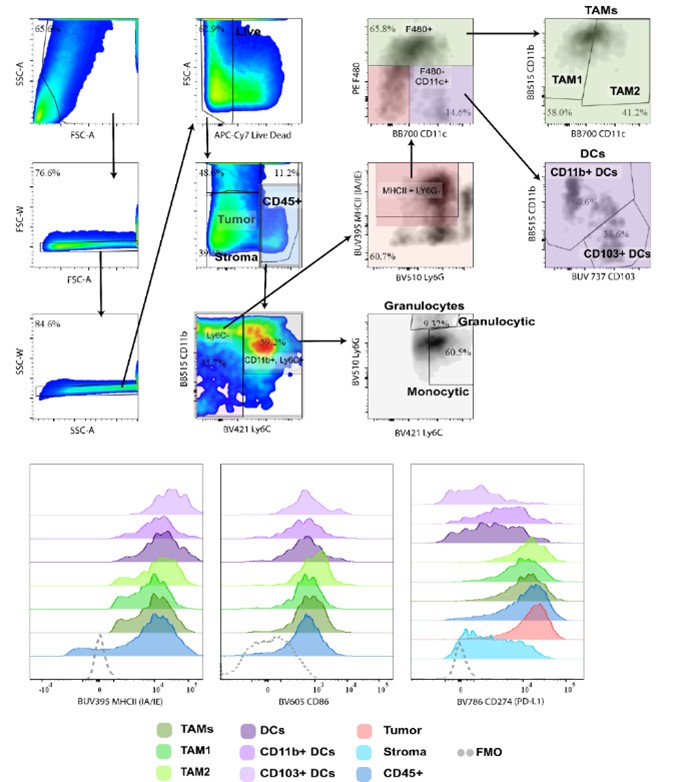

Supplement: Supplementary file 7 — Figure S7: Gating strategy for myeloid cells in the tumor and ascites (top) and spleen (bottom). [file BTM2-8-e10453-s005.zip › btm210453-sup-0009-Figure S7 top.jpg]

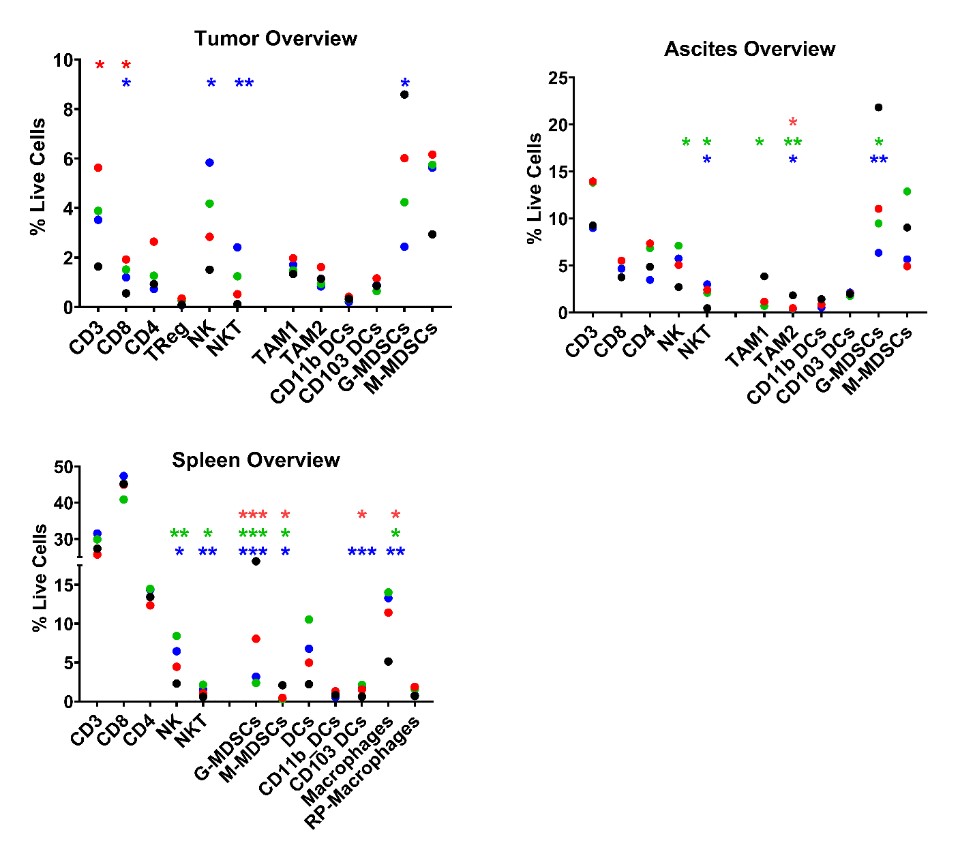

Supplement: Supplementary file 8 — Figure S8: Overall changes in immune milieu for each tissue. Statistical differences were measured by the Student's t‐test with respect to dextrose controls. *p < 0.05; **p < 0.01; ***p < 0.001. [file BTM2-8-e10453-s006.jpg]
